# Irradiation-Dependent Helium Gas Bubble Superlattice in Tungsten

**DOI:** 10.1038/s41598-019-39053-0

**Published:** 2019-02-19

**Authors:** D. J. Sprouster, C. Sun, Y. Zhang, S. N. Chodankar, J. Gan, L. E. Ecker

**Affiliations:** 1 Nuclear Science and Technology Department, Brookhaven National Laboratory, Upton, NY 11973 United States; 2 Idaho National Laboratory, Idaho Falls, ID 83415 United States; 3 National Synchrotron Light Source-II, Brookhaven National Laboratory, Upton, NY 11973 United States; 4 Department of Materials Science and Chemical Engineering, Stony Brook University, Stony Brook, NY 11794 United States

## Abstract

The implantation of noble gas atoms into metals at high gas concentrations can lead to the self-organization of nanobubbles into superlattices with symmetry similar to the metal host matrix. Here, we examine the influence of implantation parameters on the formation and structure of helium gas bubble superlattices within a tungsten host matrix to uncover mechanistic insight into the formation process. The determination of the size and symmetry of the gas bubbles was performed using a combination of small angle x-ray scattering and transmission electron microscopy. The former was demonstrated to be particularly useful in determining size and structure of the gas bubble superlattice as a function of irradiation conditions. Prior to the formation of a superlattice, we observe a persistent substructure characterized by inter-bubble spacings similar to those observable when the gas bubble superlattice has formed with very large ordering parameters. As the implantation fluence increases, the inter-bubble ordering parameter decreases, indicating improved ordering, until a superlattice is formed. Multiple implantation-specific differences were observed, including a temperature-dependent superlattice parameter that increases with increasing temperature and a flux-dependent superlattice parameter that decreases with increasing flux. The trends quantified here are in excellent agreement with our recent theoretical predictions for gas bubble superlattice formation and highlight that superlattice formation is strongly dependent on the diffusion of vacancy and implanted He atoms.

## Introduction

The energy deposited by energetic ions and neutrons in solid materials creates non-equilibrium conditions that result in supersaturated defects, such as vacancies, interstitials, and defect clusters^1^. The aggregation of these defects can cause complex microstructural transformations^2^. In the intense neutron irradiation environment of a fission nuclear reactor, in addition to the radiation damage, inert gases can be produced in the fuel and metallic fuel rod cladding^3^. Fusion materials, particularly plasma-facing components such as first wall, blanket and diverter components, will also experience extreme radiation damage and very high He fluxes^4^. As the concentration of inert gas increases in both fission and fusion materials, a high density of nanoscale bubbles form^4,5^. These bubbles have a serious impact on the mechanical and physical properties of the material, especially when they form on grain boundaries where they can lead to life-limiting embrittlement^5^. We note that in addition to bubble formation, plasma-facing materials exposed to He ions results in the formation of complex surface morphologies that are dependent on both the implantation temperature and He ion energy^6–9^.

Under specific conditions of implantation energy, temperature, fluence and ion flux, the self-organization of gas bubbles with ordered superlattices and structures similar to their host matrix have been observed^10–13^. Such superlattice structures are of importance within nuclear fuels, where they may offer large inventory capacity for fission gases, mitigate radiation damage and ultimately limit material swelling. The recent discovery of face-centered-cubic (FCC) xenon gas bubble superlattice (GBS) in body-centered-cubic (BCC) UMo challenges the widely accepted crystallographic coherency between the GBS and host matrix^14–16^. While there has been significant effort to unravel the fundamental mechanisms leading to GBS formation in the past, there is still much debate as to their nature. Currently, the most favored mechanism for formation of GBS is the anisotropic diffusion of self-interstitial atoms^13,17,18^.

Much of the analysis on bubble size, lattice symmetry and lattice parameter determination for ion implantation-induced GBS has been performed using transmission electron microscopy (TEM)^10–15^. The lack of contrast, particularly for small bubbles with incoherent or partially incoherent boundaries, makes it difficult to determine size distributions. Furthermore, size distributions generated by TEM are usually limited to several hundred bubbles. X-ray scattering methods, including small angle x-ray scattering (SAXS), intrinsically probe a significantly larger volume of material than TEM and can thus provide a more global average of size and size distribution within a sample of interest^19^. SAXS has in fact been shown to be powerful tool for studying the evolution of nanoparticles formed via ion implantation and their subsequent response to ion irradiation^20–23^. The extremely large contrast (or electron density difference |Δρ|^2^) between the dense host matrix and low-density gas bubbles make SAXS an ideal technique to determine bubble size^24^. In densely packed and concentrated systems, the distances relative to each nanoparticle (or nanobubble) become on the same order of magnitude as the nanoparticle size^24^. This leads to an additional scattering contribution in the form of a “structure factor” with the appearance of diffuse peaks and sharp Bragg peaks (if the nanoparticles are well-ordered) in measured SAXS patterns^25,26^. The location and width of these peaks can be used to determine the inter-bubble spacings and provide a measure of how ordered the bubble arrays are, respectively^27,28^.

In this article, we have performed a series of experiments to track the formation of GBS through a variety of He ion implantation conditions. We specifically report on the characterization of He GBS embedded in W host matrices. Using a common gas atom and host matrix, and systematically varying the implantation conditions (fluence, temperature and flux), we uncover mechanistic and kinetic insights into the He GBS formation.

## Methods: Experiment, Analysis and Theory

Tungsten sheets (99.95%) were purchased from Goodfellow America. The sheets were cut into multiple 3-mm-diameter discs and then twin-jet electropolished in 0.5 vol.% NaOH aqueous solution at 5 °C. The TEM samples were then implanted with 40 keV He ions at different temperatures, fluences and ion fluxes at the University of Michigan’s Michigan Ion Beam Laboratory (MIBL). The implantation parameters are summarized in Table 1. The TEM characterization of He implanted W samples were performed using a Tecnai-F30 TEM (operating at 300 keV) equipped with Gatan CCD camera.

**Table 1:** Sample matrix with implantation parameters and refined SAXS bubble size distributions and inter-bubble distances (order parameter in parentheses) and converted GBS lattice parameters.

Sample	Temp (°C)	Fluence (10^17^ ions/cm^2^)	Flux (×10^12^ ions/cm^2^/s)	Size (Å)	Inter-bubble distance (Å)	GBS lattice parameter (Å)
13*	650	1.0	6.2	23.7 ± 5.2	44.4 (19.3)	62.8
14*	500	1.0	6.2	14.4 ± 5.4	34.8 (16.2)	49.2
15	350	1.0	6.2	11.2 ± 2.5	—	—
17	500	0.6	2.7	18.1 ± 6.1	40.7 (21.3)	—
16*	500	1.0	2.7	22.8 ± 4.9	41.7 (21.7)	59.0
18*	500	2.0	2.7	23.3 ± 3.9	41.0 (14.5)	58.0
14*	500	1.0	6.2	14.4 ± 5.4	34.8 (16.2)	49.2
16*	500	1.0	2.7	22.8 ± 4.9	41.7 (21.7)	59.0
19	500	1.0	0.9	24.9 ± 5.0	41.5 (22.4)	—
*ref*. ^13^	*500*	*1*.*1*	—	*13*.*0* ± *2*.*0*	*30.1*	42.6
*ref*. ^13^	*500*	*1*.*5*	—	*16*.*1* ± *2*.*7*	*38.7*	54.7
*ref*. ^38^	*500*	*1*.*5*	—	*20*.*0* ± *2*.*0*	*43.0*	60.8
*ref*. ^38^	*600*	*1*.*0*	—	*17*.*3* ± *1*.*0*	*39.0*	55.2
*ref*. ^39^	*600*	*3*.*0*	—	*22*.*4* ± *2*.*0*	*39.0*	55.2

^*^Indicates samples that have gas bubble superlattices.

Transmission SAXS measurements on the as-implanted TEM samples were performed at the Life Science X-ray Scattering (LIX) beamline at the National Synchrotron Light Source-II^29^, using 15.50 keV X-rays (wavelength 0.7998 Å). Two-dimensional (2D) maps of the scattering intensity were collected over a 200 × 200 µm^2^ area with a two µm X-ray beam spot. The scattering intensity *I(Q)*, where *Q* is the scattering vector defined by *Q* = (*4π/λ*) × *sinθ*, *λ* is the wavelength of the incident x-rays and *θ* is half the scattering angle, was collected with Pilatus 1 M and a Pilatus 300k detectors located at sample-to-detector distances of 3861.86 and 569.09 mm, respectively. The scattering pattern from an unirradiated sample was subtracted from all He-implanted samples to isolate the signal from the bubbles and GBS. The 2D scattering maps were inspected using a custom-built software package to locate regions with the highest scattering signal. The individual SAXS and wide angle x-ray scattering detector images were then reduced in the IGOR-Pro based software package NIKA^30^ and merged together resulting in a scattering vector (*Q*) range of 0.004–1.0 Å^−1^.

Helium bubble-size distributions were determined by fitting the scattering intensity using the non-linear least squares method in IRENA^30^. For a polydisperse spherical scattering system and electron density *ρ*, embedded in a medium with electron density *ρ*_0_, the scattering intensity *I(Q)* is given by:

1 $$I(Q)={V}^{2}\times {|{\rm{\Delta }}\rho |}^{2}\times F(Q)\times S(Q)$$

Here, V is the volume of the scattering particles, |*Δρ*|^2^ is the square of the difference in electron density between the bubbles and the background medium (*Δρ* = *ρ* − *ρ*_0_), *F(Q)* is the form/shape factor, and *S(Q)* is the structure factor (inter-particle interaction parameter). Our fitting model assumes spherical bubbles (consistent with TEM investigations) with a Gaussian size distribution and an inter-precipitate structure factor to account for the high bubble densities^27,28^. Additional SAXS analysis was performed on samples that showed diffraction components due to the formation of GBS. For these samples, the 2D SAXS detector images were integrated azimuthally over a narrow range of scattering vectors (5° arc sector) in the vicinity of the diffraction peaks and analyzed with Eq. 1. This additional analysis gives a more targeted insight into the He GBS scattering component.

To aid in the interpretation of the experimental results, we performed theoretical predictions of the GBS structure following the theory developed for void superlattice^31,32^. Material parameters for W from ref.^31^ were used, and the presence of He is considered by multiplying the vacancy mobility by a trapping coefficient to account for vacancy trapping by He atoms^13^. The occupation of a vacancy by a He atom can significantly increase the vacancy migration barrier and reduce the vacancy diffusivity by several orders in the temperature range of interest here. Thus, the effective diffusivity is dictated by the fraction of vacancies not trapped by He. Without being able to exactly determine the effective diffusivity, a trapping coefficient of 0.1 is used to reflect that the vacancy mobility is reduced (compared to void superlattice formation) in the presence of He^33–36^. This trapping coefficient is expected to increase with increasing ratio of He over defect production (ion energy dependent)^13^ and implantation temperature-dependent^37^. Two defect production rates, 1.0 × 10^−5^ and 1.0 × 10^−3^ displacements-per-atom per second (dpa/s), are employed here, as they are the lower and upper bounds of the experimental defect production rates here and in previous experiments^13,38,39^.

## Results and Discussion

**Figure 1:**
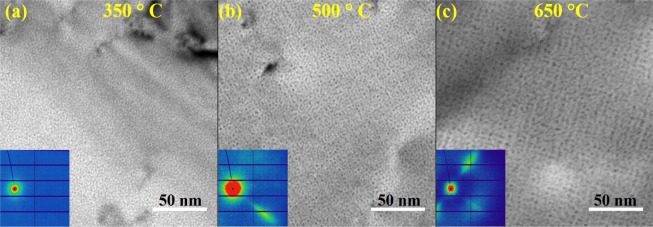
TEM images of helium implanted tungsten at (**a**) 350 °C (**b**) 500 °C (**c**) 650 °C with a beam direction close to [001]. Insets show the corresponding 2D SAXS patterns for the same samples. Diffraction peaks from gas bubble superlattices are observable in the 500 °C and 650 °C samples.

Figure 1 shows the TEM images at zone axis close to [001] as a function of implantation temperature with a common implantation fluence (1 × 10^17^ ions/cm^2^) and flux (6.2 × 10^12^ ions/cm^2^/s). The corresponding 2D SAXS images collected for the same samples are shown as insets. Qualitatively, both TEM and SAXS show the formation of small He bubbles and that ordering is only observable in the 500° and 650 °C samples, as evidenced by the pronounced diffraction component observed on the 2D SAXS images. While bubbles are clearly observable in the 350 °C sample, no GBS formation (or diffraction component) was observable in the SAXS patterns. Direct inspection of the SAXS patterns can thus be used to ascertain if a GBS has formed under specific implantation conditions.

**Figure 2:**
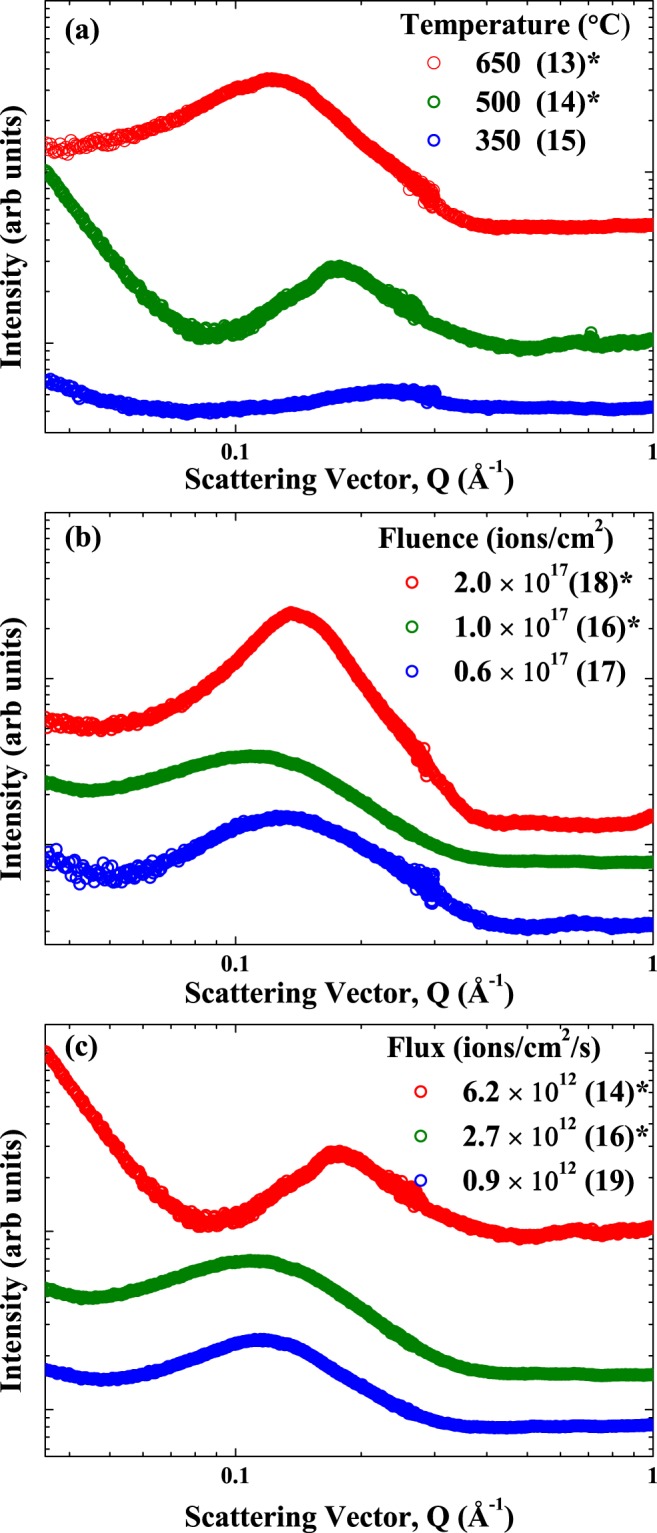
Effect of helium implantation parameters on the SAXS signal from bubbles and gas bubble superlattices in tungsten with sample ID in parentheses: (**a**) Temperature (to a fluence of 1 × 10^17^ ions/cm^2^ with a flux of 6.2 × 10^12^ ions/cm^2^/s), (**b**) Fluence (at 500 °C with a flux of 2.7 × 10^12^ ions/cm^2^/s); (**c**) Flux (at 500 °C to a fluence of 1 × 10^17^ ions/cm^2^). All samples have helium bubbles and the symbol ^*^indicates samples that have gas bubble superlattices. Samples are offset in the vertical direction for clarity. The scattering vector Q is related to characteristic length D by: Q = 2π/D.

**Figure 3:**
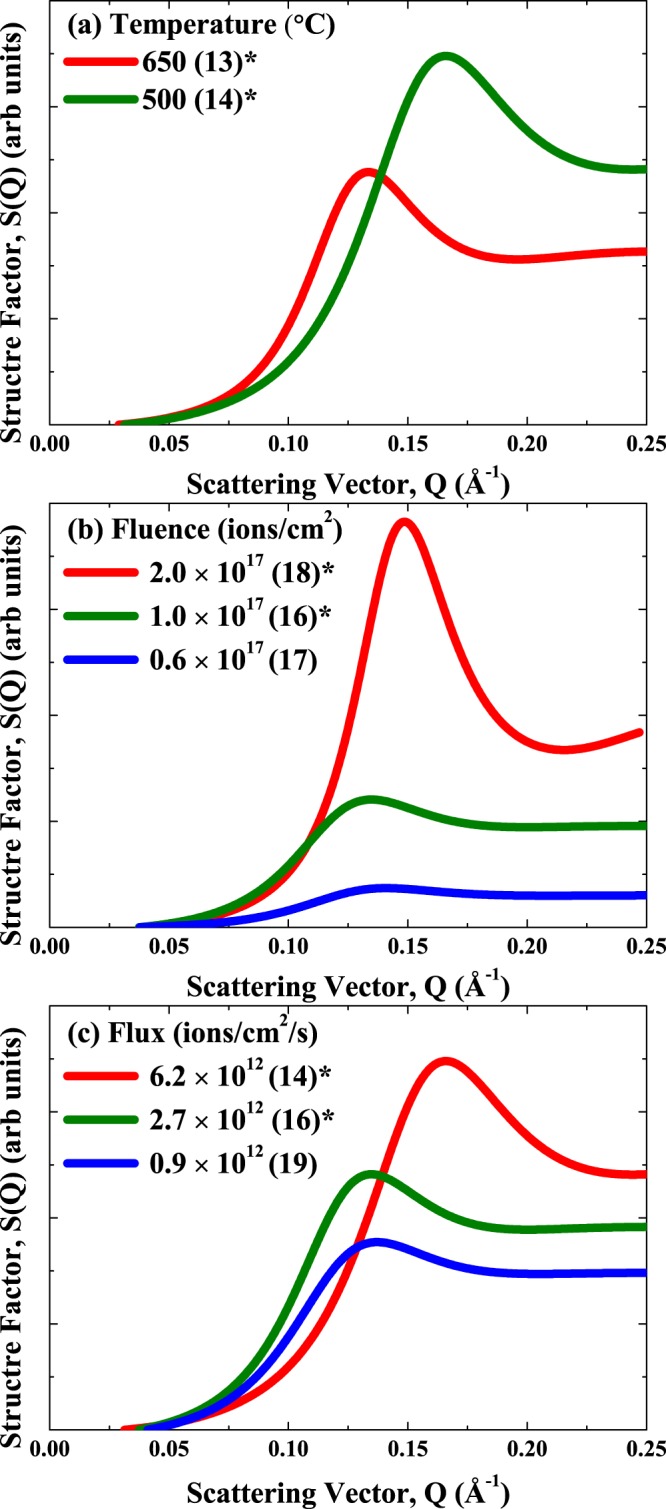
Structure factors determined from the SAXS analysis from as a function of implantation parameters: (**a**) Temperature (to a fluence of 1 × 10^17^ ions/cm^2^ with a flux of 6.2 × 10^12^ ions/cm^2^/s), (**b**) Fluence (at 500 °C with a flux of 2.7 × 10^12^ ions/cm^2^/s); (**c**) Flux (at 500 °C to a fluence of 1 × 10^17^ ions/cm^2^). All samples have helium bubbles and the symbol ^*^indicates samples that have gas bubble superlattices. Samples are offset in the vertical direction for clarity. The scattering vector Q is related to characteristic length D by: Q = 2π/D.

The integrated SAXS patterns for all implantation conditions are shown in Fig. 2. Samples that showed diffraction peaks in their SAXS images (and hence GBS formation) are indicated by an asterisk (*) as several samples did not show any evidence of diffraction in their 2D patterns. The integrated SAXS patterns for the majority of samples, irrespective of implantation conditions, show appreciable inter-bubble scattering components at *Q* ~0.04–0.35 Å^−1^. The location of these features in *Q* is inversely proportional to a characteristic distance *D* (*Q* = *2π/D*) and can be used to determine the inter-bubble spacing. This indicates that in some samples with no GBS, there is a substructure similar to a disordered array of bubbles (with no symmetry)^40^. We show below that these disordered arrays also have similar mean inter-bubble spacings and very large root-mean-square deviations in inter-bubble spacings. The inter-bubble scattering components (from the structure factor analysis) quantified from the SAXS fitting for all samples are shown in Fig. 3. The quantitative bubble sizes, inter-bubble parameter and root-mean-square deviation or degree of ordering determined from the SAXS analysis are listed in Table 1. The SAXS results show that a critical temperature, fluence, and flux and/or combinations of the implantation parameters are needed in order to promote the formation of a GBS. The bubble size and inter-bubble spacings and lattice parameters (derived from inter-bubble parameters) determined from previous He implanted W studies^13,38,39^ with similar He energies are also included for reference. The specific implantation parameter-dependent trends in the bubble size and GBS structure quantifiable from the SAXS analysis are separately discussed below.

### Temperature

For a fixed fluence (1.0 × 10^17^ ions/cm^2^) and flux (6.2 × 10^12^ ions/cm^2^/s) (Fig. 2(a)), higher implantation temperatures led to larger bubble sizes (11.2 Å at 350 °C to 23.7 Å at 650 °C). The temperature-dependent increase in bubble size is in agreement with past work, where higher mobilities of vacancies, He atoms and clusters facilitate the growth of bubbles^41–44^. Higher temperatures led to inter-bubble scattering components, as shown in Fig. 3(a), at lower-*Q* values (or larger distances, D). We note that the SAXS patterns for the 350 °C sample did not show any evidence for the formation of a GBS with no inter-scattering contributions (consistent with TEM results) and scattering more consistent with well separated bubbles^24^. The temperature-dependent increase in the inter-bubble parameter (from 34.8 Å at 500 °C to 44.4 Å at 650 °C) is in agreement with recent *in situ* TEM studies of He implanted Cu^12^ and Accelerated Kinetic Monte Carlo predictions of void superlattices in W^31^. The larger inter-bubble parameters with increasing temperature are also due to an increase in the vacancy mobility. It is interesting to note that the ordering parameter (width of the diffraction peak determined from the SAXS structure factor analysis) appears to be sharper for the 500 °C sample compared to the higher 650 °C implantation temperature. The larger order parameter and more variability in the inter-bubble parameters at higher temperature are the direct result of a less ordered GBS and lower rate of recombination of annihilating vacancies at this temperature. The ordering could thus be improved with higher fluences since the recombination with self-interstitials (which diffuse anisotropically) favors the growth of orderly arrays of bubbles.

### Fluence

For a fixed temperature (500 °C) and flux (2.7 × 10^12^ ions/cm^2^/s) condition (Fig. 2(b)), higher fluences led to larger bubble sizes. This fluence-dependent increase in He bubble size (from 18.1 Å at 0.6 × 10^17^ ions/cm^2^ to 23.3 Å at 2 × 10^17^ ions/cm^2^) in W is in agreement with past work^13,39,45^. Increasing fluence only leads to subtle changes in the position of the inter-bubble scattering peaks (Fig. 3(b)). Increasing the total fluence does result in sharper scattering peaks and lower ordering parameter (from 21.3 Å at 0.6 × 10^17^ ions/cm^2^ to 14.5 Å at 2 × 10^17^ ions/cm^2^) implying more ordered GBSs. The apparent fluence-independent GBS inter-bubble parameter quantified here with SAXS is in contrast to a recent study in W, where increasing He fluence results in a decreasing GBS inter-bubble parameter^13^. In the aforementioned study, the authors implanted He ions with different energies and fluences and examined the GBS lattice parameters as a function of He concentration/displacements per atom (dpa) (the total fluence was adjusted at each energy to reach a fixed value of 3 dpa). The different GBS lattice parameters reported by Harrison *et al*.^13^ thus reflect vacancy trapping due to different amounts of He. With a higher He/dpa ratio, the average vacancy mobility is further reduced due to the presence of more He atoms at a given dpa, leading to a smaller GBS lattice parameter. For each He/dpa rate in ref.^13^ the saturation of the GBS lattice parameter with increasing fluence is expected. In the present case, our He/dpa ratio is fixed and the inter-bubble parameter (and converted lattice parameter) saturate when an ordered superlattice forms due to the slow coarsening kinetics. Finally, the increase in bubble size and narrowing of the size distribution with increasing implantation fluence are kinetically consistent with other ion beam synthesized nanoparticle systems^42–44^.

### Flux

For a fixed temperature (500 °C) and fluence (1.0 × 10^17^ ions/cm^2^) condition (Fig. 2(c)), higher fluxes result in smaller bubble sizes (14.4 Å at 6.2 × 10^12^ ions/cm^2^/s and 24.9 Å at 0.9 × 10^12^ ions/cm^2^/s). The trends in bubble size with flux are consistent with other ion beam synthesized nanoparticle studies, where higher fluxes lead to smaller nanoparticle sizes^42–44^. The implantation flux has a significant effect on the structure factor from the SAXS analysis, with high flux resulting in smaller inter-bubble parameters (34.8 Å at 6.2 × 10^12^ ions/cm^2^/s to 41.5 Å at 0.9 × 10^12^ ions/cm^2^/s) (Fig. 3(c)). Higher flux also results in smaller ordering parameters (16.2 Å at 6.2 × 10^12^ ions/cm^2^/s to 22.4 Å at 0.9 × 10^12^ ions/cm^2^/s) and sharper diffraction peaks. The effect of flux on the GBS lattice parameter and the amount of ordering quantified experimentally here are in agreement with our recent theoretical results for void superlattices^31,32^.

### Comparison with theory

**Figure 4:**
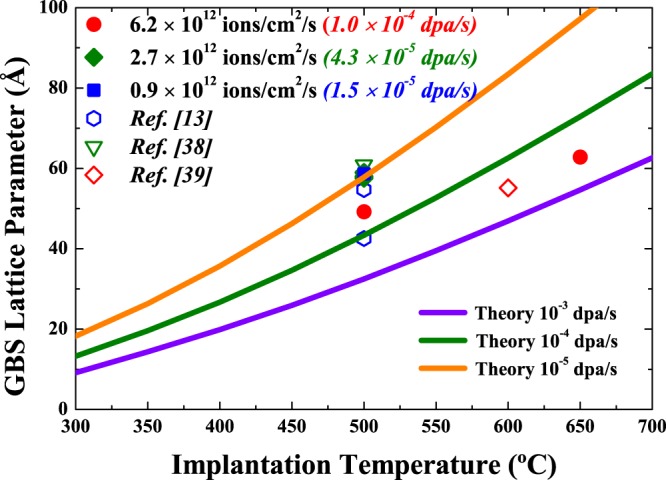
GBS lattice parameter as a function of implantation temperature. The solid curves show the theoretical predictions for GBS lattice parameter. The filled symbols are from experiments reported here and open symbols are from refs^13,38,39^.

The theoretically predicted and experimentally determined GBS lattice parameters are shown in Fig. 4. From our theory, the GBS lattice parameter is predicted to increase with vacancy mobility and steadily increase with temperature. We also predict that the GBS lattice parameter will have a flux dependence and decrease with an increase in defect production and annihilation rate. The GBS lattice constant (a) was estimated by converting the inter-bubble distance (d) of gas bubbles on (110) planes by a = √2*d. The experimentally determined GBS lattice parameters are qualitatively within the theoretically predicted curves. As previously stated, increasing the implantation temperature effectively increases the vacancy mobility (diffusivity), leading to an increase in the GBS inter-bubble distance/lattice parameter. The temperature-dependent trends experimentally determined and theoretically predicted here are consistent with results in refs^31,32^ for void superlattices. Figure 4 shows that at a fixed temperature, increasing the defect production rate from 10^−5^ to 10^−3^ dpa/s led to a decrease in the GBS lattice parameter. The representative defect production rates shown here are used to cover typical ion irradiation conditions used in this and previous work. The same trend is experimentally quantified here for samples implanted at 500 °C with ion fluxes of 0.9 to 6.2×10^12^ ion/cm^2^/s. From our theory, the formation of a GBS requires the accumulation of vacancies. We have previously shown that a similar critical vacancy concentration is needed to induce the formation of a void superlattice^31,32^. The results here suggest that both GBS and void superlattice formation require the net defect production rate to outweigh annihilation events (via recombination and sink absorption). Additionally, increasing the ion flux led to an enhanced recombination (relative to sink absorption) and improved ordering, consistent with the SAXS derived ordering parameter results in Table 1. In general, the effects of increasing flux are similar to that of decreasing temperature. We make note that our theory also predicts the formation of disordered GBSs (prior to complete ordering), with similar trends in the inter-bubble and ordering parameters^32^. The agreement between the experiment and theory across the implantation conditions examined here (and by others) highlights the similarity of void superlattices and GBS and that they may potentially be unified within a similar framework (provided that an appropriate trapping coefficient is considered).

## Conclusions

In summary, we have investigated the effects of implantation parameters (temperature, fluence and flux) on the formation of He GBS in W. We have identified several implantation- specific differences in both He bubble size and GBS structure. These include a temperature-dependent increase in bubble size (11.2 Å at 350 °C to 23.7 Å at 650 °C) and inter-bubble spacing (from 34.8 Å at 500 °C to 44.4 Å at 650 °C); an improved ordering with increasing He fluence; and a reduced bubble size with increasing ion flux (14.4 Å at 6.2 × 10^12^ ions/cm^2^/s and 24.9 Å at 0.9 × 10^12^ ions/cm^2^/s). The SAXS technique was effective in characterizing both evolution of the bubbles and GBS and could potentially be utilized in future studies to determine critical information needed in similar metallic systems. Our results are in excellent qualitative agreement with our recent theoretical predictions for void superlattices formation in W and indicate that the GBS formation mechanism is similarly dependent on the mobility of vacancies and He interstitials. Our systematic experimental study and theoretical predictions have shed light on the fundamental mechanisms responsible for He GBS formation in W. Finally, this methodology could potentially be utilized to predict/control defect evolution in other relevant material systems where similar implantation-induced modifications are observed.
